# Lethal Necrotizing Pneumonia Caused by an ST398 *Staphylococcus aureus* Strain

**DOI:** 10.3201/eid1608.100317

**Published:** 2010-08

**Authors:** Jean-Philippe Rasigade, Frederic Laurent, Philippe Hubert, François Vandenesch, Jerome Etienne

**Affiliations:** Hospices Civils de Lyon, Lyon, France (J.P. Rasigade, F. Laurent, J. Etienne, F. Vandenesch); Assistance Publique–Hôpitaux de Paris, Paris, France (P. Hubert)

**Keywords:** Community-acquired infections, pneumonia, bacteria, Staphylococcus aureus, methicillin-resistant, Panton-Valentine leukocidin, humans, letter

**To the Editor:** Several recent studies have shown massive colonization of livestock (especially pigs) and livestock workers by methicillin-resistant *Staphylococcus aureus* (MRSA) in western Europe, Canada, and the United States (*1**,**2*). Livestock MRSA isolates belong almost exclusively to a single sequence type, ST398. Evidence of zoonotic and interhuman transmission of methicillin-resistant and methicillin-susceptible variants of this hitherto unusual sequence type was recently reported (*1**,**3*). *S. aureus* ST398 infections in humans with or without a history of contact with livestock include bacteremia, endocarditis, ventilator-associated pneumonia, and wound infections, none of which involve the expression of specific toxins. Indeed, ST398 isolates are negative for all major virulence factors, with the exception of some rare isolates that harbor the genes that encode the Panton-Valentine leukocidin (PVL) (*1*), a toxin that is usually associated with community-acquired MRSA (*4*). We report a case of lethal necrotizing pneumonia caused by a PVL-positive methicillin-susceptible ST398 *S. aureus* isolate.

A previously healthy 14-year-old girl came to the emergency room with influenza-like illness, cough, fever, and a 2-day history of severe abdominal pain. She was given intravenous antibacterial chemotherapy with cefotaxime and amikacin. An exploratory laparotomy showed no signs of abdominal disease. Immediately after surgery, acute respiratory distress syndrome with hemodynamic instability developed in the patient; mechanical ventilation and inotropic support were required. A chest radiograph showed bilateral pulmonary infiltrates and pleural effusion. *S. aureus* was isolated by bronchoalveolar lavage fluid and blood culture, and staphylococcal necrotizing pneumonia was diagnosed. Clinical features, including the preceding influenza-like illness, were highly consistent with those previously reported (*5*). However, viral cultures and immunofluorescence assays were negative for all common respiratory viruses, and, although the patient had positive serologic test results for influenza B virus, antibody titers were too low to affirm influenza B infection. Severity factors were present (*5*), including leukopenia, airway bleeding, and multiorgan failure. She died 6 days after symptom onset, with refractory shock and respiratory failure caused by bilateral pneumothorax. The *S. aureus* strain, which was susceptible to all tested antimicrobial agents except macrolides, was *agr*1/ST398, *spa*-type t571 and nontypeable by *Sma*I pulsed-field gel electrophoresis, which showed its relatedness to livestock-associated strains. The origin of the infection could not be determined. The presence of the genes encoding PVL was confirmed by PCR.

Thus, the spread of *S. aureus* ST398 among livestock is a matter of increasing concern because strains of this sequence type were able to acquire PVL genes and cause necrotizing pneumonia in a young immunocompetent patient. Transmission control and surveillance efforts are urgently needed to prevent further spread of such strains.
